# Ventricular Fibrillation Cardiac Arrest in African American Male with Apical Hypertrophic Cardiomyopathy

**DOI:** 10.7759/cureus.3267

**Published:** 2018-09-07

**Authors:** Giselle Volney, Habiba Wada, Michael Tatusov

**Affiliations:** 1 Internal Medicine, Ross University School of Medicine, Roseau, DMA; 2 Internal Medicine, Ross University School of Medicine, Bowie, USA; 3 Medicine/Trauma and Critical Care, University of Maryland School of Medicine, Baltimore, USA

**Keywords:** ace of spades, apical hypertrophic cardiomyopathy, ventricular fibrillation

## Abstract

Apical hypertrophic cardiomyopathy (AHCM) is a rare form of non-obstructive hypertrophic cardiomyopathy. It is rarely reported in African American patients, and more commonly reported in Japanese patients. AHCM involves hypertrophy of the apex of the left ventricle. It is considered to have a benign prognosis in terms of cardiovascular mortality, however arrhythmias and sudden cardiac death have been reported. We report a case of a 49-year-old African American male with a history of hypertension, who presented to the emergency department after in field defibrillation for ventricular fibrillation cardiac arrest with return of spontaneous circulation after 10 minutes of cardiopulmonary resuscitation. Features of left ventricular hypertrophy and deep T-wave inversions in V3-V6 were noted on a 12-lead electrocardiogram which were suggestive of AHCM. Left heart catheterization with left ventriculography and coronary angiography confirmed the diagnosis of AHCM with the classic “ace of spades” sign. This case highlights the rare occurrence of AHCM with ventricular fibrillation cardiac arrest in an African American male, treated with hypertension management, aspirin, atorvastatin and automated implantable cardioverter-defibrillator placement.

## Introduction

Apical hypertrophic cardiomyopathy (AHCM) is a rare variant of hypertrophic cardiomyopathy (HCM) [1]. The prevalence of HCM in the general population has been estimated at 0.2%, of which an estimated 95% of patients have asymmetric hypertrophy of the LV septum, and 25% of patients have associated LV intracavitary obstruction [2]. AHCM is estimated to occur in about 3% of the HCM cases within the United States of America (USA), and 15% of HCM cases in Japan, where the distal portion of the left ventricular wall is thickened [1]. This form of hypertrophic cardiomyopathy is non-obstructive. Clinically, the presentation of AHCM may vary, and an electrocardiogram (ECG) and echocardiography are usually used for initial diagnosis [1]. Characteristic findings on ECG of patients with AHCM include large QRS complexes in the precordial leads, particularly in lead V4, with rightward superior, and posterior shift of the T-wave vector. Automated interpretation of printed reports of ECGs may show probable ischemia, left ventricular hypertrophy (LVH), and/or left ventricular anterolateral repolarization abnormalities consistent with left ventricular strain pattern [3]. Transthoracic echocardiography often detects AHCM if suspicion is high, however AHCM is frequently missed by echocardiography as it is uncommon and the degree of suspicion may be low [4].

## Case presentation

A 49-year-old African American male presented to our hospital center after ventricular fibrillation cardiac arrest with return of spontaneous circulation achieved after 10 minutes of cardiopulmonary resuscitation and defibrillation by emergency services. Personal cardiovascular risk factors included untreated hyperlipidemia and hypertension. Cardiac past medical history included one episode of diaphoresis and palpitations, four years prior to this admission. Per the patient, workup at that time at another local hospital revealed an unspecified arrhythmia and cardiac hypertrophy, but the patient did not follow up. The patient denied a family history of recurrent syncope or unexplained cardiac death, but reported unspecified cardiac hypertrophy and unspecified arrhythmia in one brother, and coronary artery disease in mother and brother.

On arrival to the emergency department, the patient was asymptomatic. Clinical examination showed a blood pressure of 135/67, with irregular heartbeat of 72 beats per minute, decreased heart sounds and soft systolic murmur but no S4 on cardiac auscultation. Troponin-T was minimally elevated at 0.021 ng/mL. Lipid panel was deranged – cholesterol 239 mg/dL, triglycerides 149 mg/dL, low-density lipoprotein cholesterol 170 mg/dL, and high-density lipoprotein cholesterol 48 mg/dL. Transaminitis (aspartate aminotransferase 504 unit/L, alanine aminotransferase 332 unit/L), elevated creatinine 1.5 mg/dL and anion gap were noted on laboratory studies. Other labs were unremarkable including normal white blood cell count, hemoglobin and thyroid-stimulating hormone.

**Figure 1:**
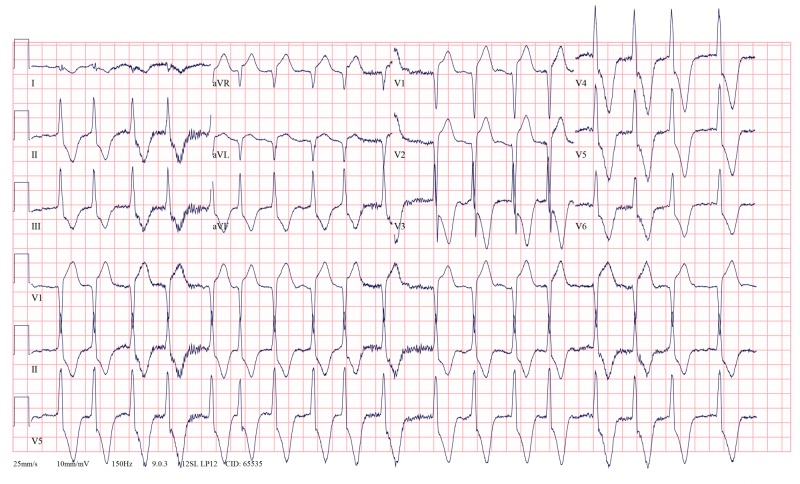
Deep T wave inversions in V3-V6 in a patient with apical hypertrophic cardiomyopathy (AHCM).

**Figure 2:**
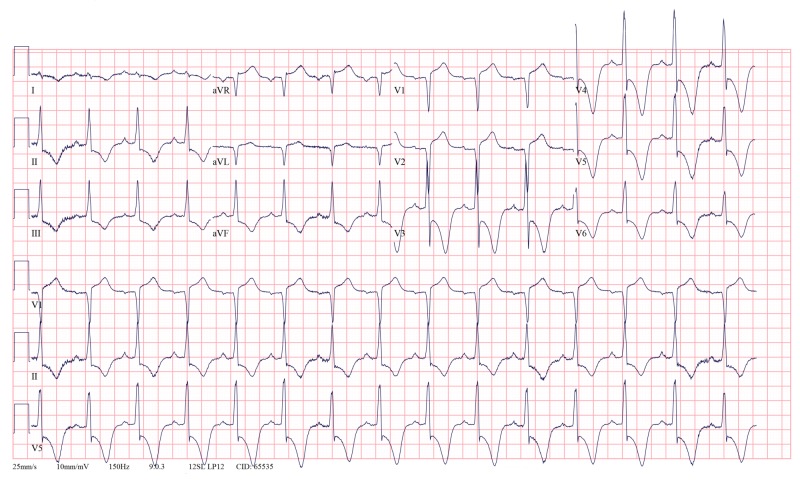
Persistence of deep T wave inversions in V3 to V6 of a patient with AHCM. AHCM: Apical hypertrophic cardiomyopathy

**Figure 3:**
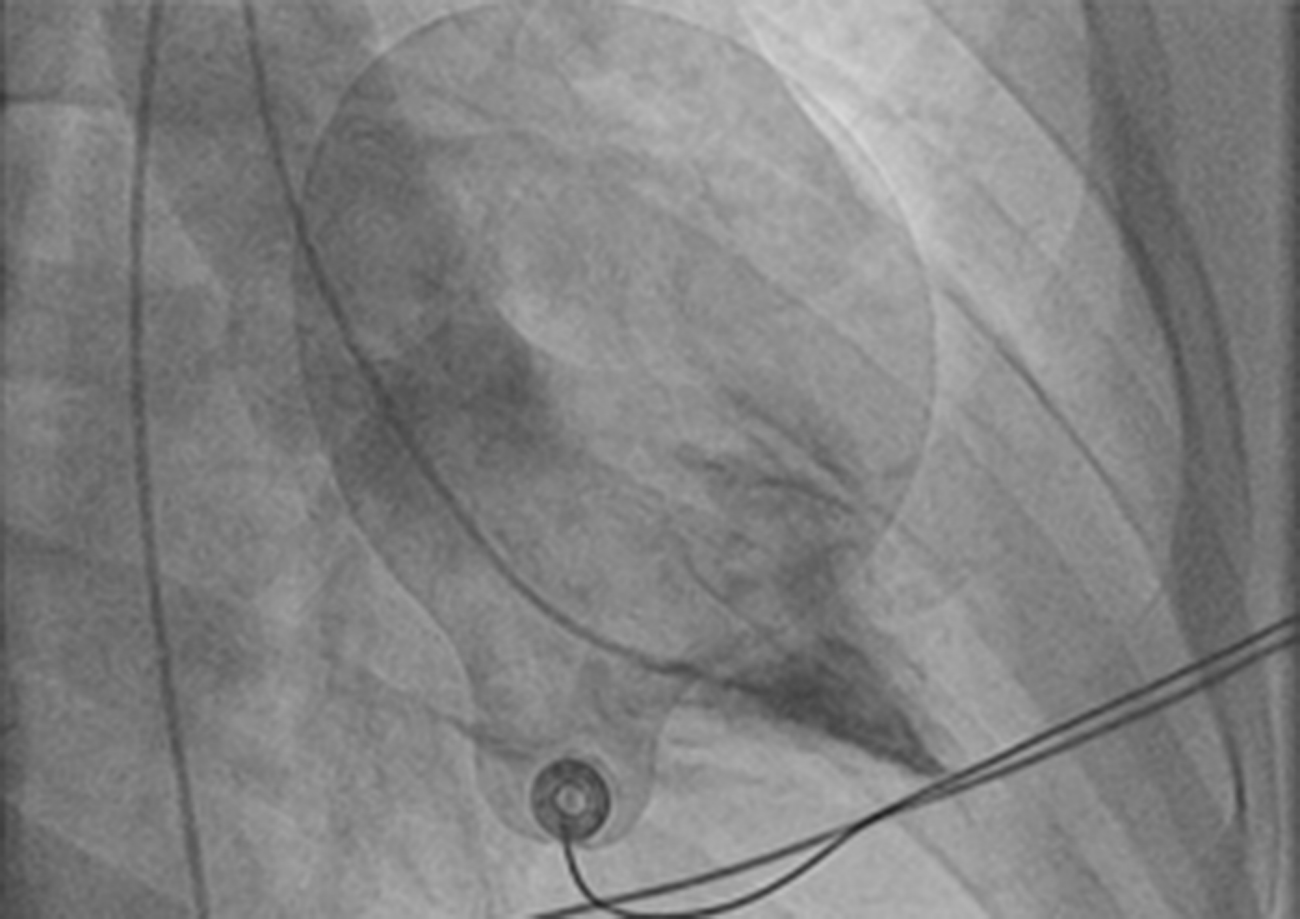
Classic “Ace of Spades” sign on ventriculogram of a patient with AHCM. AHCM: Apical hypertrophic cardiomyopathy

Serial 12-lead ECGs showed deep T inversions in V3-V6 and early repolarization in V1 and V2 leads (Figures 1, 2). On arrival, the patient was also in atrial fibrillation with rapid ventricular response (Figure 1), which resolved with intravenous Amiodarone. Septal infarct of undetermined age, possible inferior subendocardial injury, possible anterolateral subendocardial injury, and prolonged QT were also reported on automated interpretation of ECG. The patient underwent cardiac catheterization, which showed patent coronary arteries, however, the classic ace of spades sign was seen on ventriculogram (Figure 3). Initial transthoracic echocardiogram without contrast was interpreted as normal left ventricular wall thickness, with an ejection fraction of 70% and no wall motion abnormalities.

The patient was treated with aspirin 81 mg daily, carvedilol 6.25 mg twice a day, hydrochlorothiazide 25 mg daily, atorvastatin 40 mg daily, and an automated implantable cardioverter-defibrillator (AICD) for secondary prevention of another cardiac arrest. The patient was asymptomatic up to discharge three days after admission. Liver function enzymes (aspartate aminotransferase and alanine aminotransferase) trended down to 65 unit/L and 199 unit/L, respectively. Creatinine also normalized to 1.1 mg/dL.

## Discussion

AHCM is a rare non-obstructive morphological variant of HCM [5]. AHCM is rarely reported in African American patients [6], and is most prevalent in the Japanese population. The prevalence of AHCM among HCM patients in Japan was 15%, whereas in the USA the prevalence was only 3% [1, 7]. In AHCM, left ventricular (LV) wall thickening is confined to the apex (isolated asymmetric apical hypertrophy), unlike classical HCM where left ventricular hypertrophy occurs primarily in interventricular septum, often resulting in decreased chamber size and outflow obstruction [8]. AHCM, however, can co-exist with hypertrophy of the interventricular septum, classified as mixed AHCM [5].

AHCM is most commonly sporadic, however, an autosomal dominant inheritance pattern has been reported within some families [5]. Mutations in cardiac sarcomere protein genes like β-cardiac myosin heavy chain (MYH7), cardiac myosin binding protein C (MYBPC3), cardiac troponin T (TNNT2) and I (TNNI3), most commonly associated with HCM can result in multiple cardiac morphologies including AHCM [5, 9]. However, only a few sarcomere gene defects like cardiac actin Glu101Lys consistently produce AHCM [5].

Patients with AHCM are often asymptomatic, and may be undiagnosed or diagnosed incidentally. The condition has a relatively benign prognosis, however, non-fatal cardiac events may occur in about 33% of patients and the annual cardiovascular mortality of patients with AHCM has been estimated at 0.1% [10]. Symptomatic patients are commonly middle aged, and are more commonly male [1]. Patients may present with complaints of chest pain, palpitations, dyspnea and syncope. Patients may also present with atrial fibrillation, myocardial infarction, thromboembolism and congestive heart failure [1, 11]. Non-sustained ventricular tachycardia and sudden cardiac death are rare in patients with AHCM, but have also been reported [12]. The risk of sudden cardiac death or sustained ventricular tachycardia in patients with HCM increases by five times if risk factors such as a family history of sudden cardiac death, history of syncope, LV hypertrophy greater than 30–35 mm, or high-risk genetic mutations are present [2]. An abnormal blood pressure response to exercise and asymptomatic non-sustained ventricular tachycardia are other high risk factors [1]. Despite AHCM lacking the obstructive mechanism behind sudden cardiac death in hypertropic obstructive cardiomyopathy, fatal complications from AHCM often occur, due to apical aneurysm or cardiac arrest [1, 11]. The incidence of patients with AHCM developing apical aneurysms has been estimated at 10% to 20%, and ventricular arrhythmia often originates from the apical aneurismal segment [2]. Unlike patients with HCM, where ejection fraction can be reduced substantially and patients often present with syncope or sudden death, AHCM is non-obstructive, and ejection fraction is not typically affected [8]. Patients with AHCM maintain normal stroke volume even with decreased preload [10]. Patients with advanced age, hypertension, diabetes, baseline atrial fibrillation have a poorer prognosis [5].

AHCM is primarily diagnosed by history, ECG and diagnostic imaging studies [13]. Many patients with AHCM present with large QRS complexes in the lateral leads suggestive of LVH, without a history of hypertension [13]. Deep T-wave inversions in precordial leads are characteristic, and T wave inversions can be >10 mm. A minority of patients may have a normal ECG [10]. Transthoracic echocardiogram is often used as another initial study in patients with AHCM, and can show hypertrophy of the LV apex [14]. ECG and echocardiogram without contrast, however, are insufficient for a diagnosis of AHCM [1]. AHCM may be missed on echocardiography, and AHCM may have been underreported due to overreliance on echocardiography [15]. Definitive diagnosis of ACHM requires imaging modalities such as cardiac magnetic resonance and contrast echocardiography [1]. In ACHM, the thickening of the apex leads to narrowing of the ventricular chamber near the apex. The contour of the left ventricular chamber has a shape similar to a “spade” called the “Ace of Spades” sign as a result [1]. Cardiac magnetic resonance imaging without contrast is capable of highlighting the outline of the ventricular chamber and clearly displays thickening of the apical myocardium [15]. Contrast echocardiography (ventriculogram) is also useful in outlining the contours of the ventricular chamber. The contour of the chamber highlighted by contrast will show narrowing near the apex [1]. Diagnostic criteria suggested for AHCM, based on an echocardiogram or magnetic resonance imaging, include asymmetric LVH mainly at the LV apex – the thickness of the apical wall ≥ 15 mm and a ratio of maximal apical to posterior wall thickness ≥1.5 [10]. The thickness of the wall can range from mild to severe (>28 mm) [15].

As with the treatment of obstructive HCM, where the use of beta-blockers and calcium channel blockers is a mainstay of therapy [16], symptomatic AHCM patients may also benefit from the negative inotropic effects of beta blockers and calcium channel blockers like verapamil [6, 12]. Unlike HCM patients with an elevated outflow gradient at rest, where a reduction of LV afterload can worsen symptoms [17], the reduction of afterload with angiotensin-converting enzyme inhibitors has also been recommended for symptomatic patients with AHCM [6]. For patients who present with atrial fibrillation and ventricular arrhythmias, antiarrhythmics such as amiodarone and procainamide can be used [16]. Patients with AHCM who experienced cardiac arrest or ventricular tachycardia have been treated with an AICD and verapamil with good outcome [12]. Patients with apical aneurysms and arrhythmias refractory to antiarrhythmic drug therapy require AICD placement [2]. Avoidance of agents that may prolong the QT interval is also recommended, as QT prolongation may be seen in AHCM patients [2]. Overall, however, the response to medical therapy of symptomatic patients with AHCM may be poorer than that for patients with obstructive HCM [18]. Further, unlike HCM patients, where surgical myectomy and alcohol septal ablation have evolved as standard modalities to treat HCM patients refractory to medical management [17], use of such techniques in patients with AHCM is less often reported, but are used. Alcohol ablation has been used as palliative treatment in mixed AHCM – with severe mid-LV cavity gradient and symptoms refractory to medical management, and to eliminate monomorphic ventricular tachycardia after failed radiofrequency ablation [3]. Apical myectomy and heart transplant have been considered as options for the subset of AHCM patients with severe heart failure symptoms refractory to medical therapy [6]. Such patients often have diastolic heart failure with low cardiac output. Unlike with HCM where myectomy is often subaortic to relieve the LV outflow tract obstruction, apical myectomy is used as a means of LV cavity enlargement and augmentation of LV end-diastolic volume, with overall improvement in functional status [18]. Overall, close follow-up of AHCM patients is recommended as although a generally benign prognosis, some patients may rarely develop sudden life-threatening complications [10].

## Conclusions

With increasing numbers of patients with AHCM being reported among non-Japanese population, recognizing the clinical features of AHCM is valuable for patient management. AHCM should be suspected in patients with deep inverted T waves in the precordial leads on EKG, and other signs of LVH on EKG. A normal echocardiogram does not rule out AHCM, as it is commonly missed. Although typically benign, there are serious cardiac complications which may result from AHCM, and treatment may consist of negative inotropes, antiarrhythmics, angiotensin-converting enzyme inhibitors and AICD placement when warranted. More invasive techniques have been reserved for a subset of patients with symptoms refractory to medical therapy. Periodic lifelong follow-up is recommended and family screening should be considered even for asymptomatic patients as significant cardiovascular events can happen. Increased understanding among physicians of this cardiac abnormality may help guide physicians in diagnosing, treating, and monitoring the cardiac complications of patients.
